# Comparative proteomic characterization of seminal plasma and sperm proteins associated with reproductive function in the Indo-Pacific bottlenose dolphin (Tursiops aduncus)

**DOI:** 10.14202/vetworld.2026.3823-3835

**Published:** 2026-08-27

**Authors:** Mokhamad Fahrudin, Brilla Widya Witri, Iis Arifiantini, Wahono Esthi Prasetyaningtyas, Agustin Indrawati, Ni Wayan Kurniani Karja

**Affiliations:** 1 Division of Anatomy, Histology and Embryology, School of Veterinary Medicine and Biomedical Sciences, IPB University, Bogor 16680, Indonesia.; 2 Veterinary Biomedical Science Study Program, School of Veterinary Medicine and Biomedical Sciences, IPB University, Bogor 16680, Indonesia.; 3 Division of Reproduction and Obstetrics, School of Veterinary Medicine and Biomedical Sciences, IPB University, Bogor 16680, Indonesia.; 4 Wersut Seguni Indonesia Animal Park Klampok, Sendang Sikucing, Rowosari, Kendal Regency, Central Java 51354, Indonesia.

**Keywords:** assisted reproductive technology, cetacean reproduction, Indo-Pacific bottlenose dolphin, LC–MS/MS, oxidative phosphorylation, proteomics, seminal plasma, sperm proteome

## Abstract

**Background and Aim:**

Proteomic characterization of semen provides molecular insights into reproductive function beyond conventional semen evaluation. Although the seminal plasma and sperm proteomes of the common bottlenose dolphin (*Tursiops truncatus*) have been reported, corresponding information for the Indo-Pacific bottlenose dolphin (*Tursiops aduncus*) is lacking. This study aimed to characterize the protein composition of seminal plasma and sperm in *T. aduncus* to identify proteins and biological pathways associated with reproductive function.

**Materials and Methods:**

Semen samples were collected from two healthy adult captive male *T. aduncus* exhibiting >70% sperm motility. Equal volumes of semen from both animals were pooled, and seminal plasma and sperm fractions were separated by centrifugation. Proteins were extracted, separated by sodium dodecyl sulfate-polyacrylamide gel electrophoresis, and identified using liquid chromatography-tandem mass spectrometry. Protein identification was performed with Proteome Discoverer 2.5 using a 1% false discovery rate. Functional annotation was conducted using Gene Ontology enrichment and Search Tool for the Retrieval of Interacting Genes/Proteins protein-protein interaction analyses.

**Results:**

A total of 199 proteins were identified, including 84 proteins in seminal plasma and 119 in sperm, with only four proteins shared between the two fractions. Seminal plasma proteins were predominantly enriched in carbohydrate and lipid catabolism, proteolysis, and regulation of endopeptidase activity, indicating roles in maintaining the extracellular environment, protecting sperm, and supporting post-ejaculatory survival. In contrast, sperm proteins were significantly enriched in oxidative phosphorylation, aerobic respiration, mitochondrial adenosine triphosphate synthesis, respiratory electron transport, spermatogenesis, sperm flagellum organization, and motility. Protein interaction analysis demonstrated a highly interconnected mitochondrial network in sperm, whereas seminal plasma proteins formed a more dispersed regulatory network. These findings indicate distinct functional specialization of the two semen fractions and suggest that oxidative phosphorylation represents the principal energy-producing pathway supporting sperm function in this marine mammal.

**Conclusion:**

This study provides the first comprehensive proteomic characterization of seminal plasma and sperm in the Indo-Pacific bottlenose dolphin. The identified proteins and enriched biological pathways advance understanding of cetacean reproductive physiology, reveal molecular adaptations associated with mitochondrial energy metabolism, and establish a valuable resource for developing fertility biomarkers and assisted reproductive technologies to support the conservation and reproductive management of this Near Threatened species.

## INTRODUCTION

Dolphins and other small cetaceans are apex marine predators that play essential roles in shaping marine community structure, biological interactions [1], and ecosystem stability [2]. Among cetaceans, the Indo-Pacific bottlenose dolphin (*Tursiops aduncus*) is one of the most widely distributed species, inhabiting shallow coastal and neritic waters throughout the Indian Ocean, Australian waters, and Southeast Asia [3]. Its coastal distribution makes it particularly vulnerable to environmental degradation, direct exploitation, and fisheries-related threats [4], thereby increasing its risk of population decline. Until recently, *T. aduncus* was classified as Data Deficient on the International Union for Conservation of Nature (IUCN) Red List of Threatened Species; however, its conservation status was revised to Near Threatened in 2019 [3]. IUCN Red List assessments are updated periodically and may not fully reflect recent changes in population status. Furthermore, the IUCN category represents the global conservation status of the species and may not accurately reflect the status of local populations, including those in Indonesian waters. Therefore, preventing further population decline remains essential for preserving marine ecosystems.

Integrating artificial insemination (AI) with genome resource banking offers a promising strategy for the genetic management of bottlenose dolphins [5]. Successful AI requires a comprehensive understanding of species-specific reproductive physiology, particularly male reproductive function, to support captive breeding programs and the effective use of genetic resources. Male fertility is conventionally evaluated by semen analysis, including sperm concentration, motility, morphology, and viability, which remains the cornerstone of reproductive assessment in mammals, including cetaceans [6, 7]. However, conventional semen analysis does not fully evaluate sperm functional competence [8] and provides limited insight into the subcellular and molecular mechanisms underlying fertilization and reproductive success [9]. Fertilization depends not only on semen quality but also on a series of highly coordinated functional events, including sperm transport, hyperactivation, the acrosome reaction, penetration of the cumulus-oophorus and zona pellucida, and fusion with the oocyte [10]. Semen consists of two major components: spermatozoa and seminal plasma. Spermatozoa are produced in the seminiferous tubules of the testes, whereas seminal plasma is secreted by the accessory sex glands and excurrent ducts. In bottlenose dolphins, seminal plasma is derived primarily from the prostate, the only accessory sex gland described in cetaceans [11].

Proteomic analysis provides a powerful approach for evaluating reproductive potential by identifying proteins and molecular pathways associated with sperm function, fertility, and semen quality [12]. Proteomics has emerged as an important tool for elucidating the composition and biological functions of spermatozoa and seminal plasma, as well as identifying proteins and pathways associated with male infertility [13]. Proteomic approaches have been applied to the sperm of several aquatic species, including teleost fish and marine mammals [14–16]. However, proteomic information remains limited for bottlenose dolphins. Although the seminal plasma and sperm proteomes of *T. truncatus* have been characterized [16], corresponding data for *T. aduncus*, now recognized as a distinct species, are unavailable. This study therefore provides the first comprehensive proteomic characterization of seminal plasma and sperm in *T. aduncus*, offering species-specific insights into its reproductive physiology. Furthermore, this study explores seminal proteomic profiles in relation to adaptations of energy metabolism to a carbohydrate-limited marine environment, while establishing a foundational resource for developing fertility biomarkers and assisted reproductive technologies for vulnerable cetaceans.

Proteomic studies have substantially improved the understanding of sperm function and fertility in domestic animals and several aquatic species by identifying proteins and biological pathways associated with reproductive competence. However, comparable molecular information is extremely limited for cetaceans. To date, proteomic characterization has been reported only for *T. truncatus* [16], whereas the seminal plasma and sperm proteomes of *T. aduncus* remain unexplored despite its recognition as a distinct species with unique ecological and evolutionary characteristics. The lack of species-specific proteomic information limits understanding of the molecular mechanisms underlying sperm function, energy metabolism, and reproductive physiology in *T. aduncus*. Furthermore, this knowledge gap hinders the identification of candidate biomarkers for semen quality and fertility assessment and restricts the development of evidence-based assisted reproductive technologies and genetic conservation strategies for this Near Threatened cetacean species.

This study aimed to comprehensively characterize the protein composition of seminal plasma and sperm in the *T. aduncus* using liquid chromatography-tandem mass spectrometry (LC–MS/MS). The identified proteins were functionally annotated through Gene Ontology (GO) enrichment and protein-protein interaction analyses to elucidate the biological processes and molecular pathways associated with male reproductive function. The study also sought to establish a species-specific proteomic reference that could facilitate the discovery of fertility-associated biomarkers and provide a molecular foundation for future reproductive management, assisted reproductive technologies, and conservation programs for *T. aduncus*.

## MATERIALS AND METHODS

### Ethical approval

All procedures involving *T. aduncus*, including animal handling and semen collection, were reviewed and approved by the Animal Ethics Committee of the School of Veterinary Medicine and Biomedical Sciences, IPB University, Bogor, Indonesia (Approval No. 092/KEH/SKE/VIII/2023). The study was conducted in accordance with the institutional guidelines and ethical principles governing the use, care, and welfare of animals in research. Particular attention was given to minimizing animal discomfort and stress during all procedures.

The study involved two healthy adult male *T. aduncus*, aged 11 and 18 years and weighing approximately 110 and 105 kg, respectively, maintained under identical environmental, nutritional, and reproductive manage-ment conditions at Wersut Seguni Indonesia Animal Park, Kendal Regency, Central Java, Indonesia. Semen collection was performed only from animals previously trained and conditioned for the procedure. Each dolphin voluntarily positioned its ventral surface above the water level in response to trainer commands, after which manual penile stimulation was performed until ejaculation. The ejaculate was collected directly into a sterile collection bottle and immediately transported to the laboratory. No sedation or invasive procedures were used during semen collection, and all procedures were performed by trained personnel with measures to minimize stress and safeguard animal welfare throughout the study.

### Study period and location

The study was conducted on 26 January 2024. Semen samples were collected from captive *T. aduncus* housed at Wersut Seguni Indonesia Animal Park, Kendal Regency, Central Java, Indonesia. Laboratory analyses were subsequently performed according to the study protocol.

### Study design

This descriptive proteomic study aimed to characterize the protein composition of seminal plasma and sperm. Equal volumes of semen collected from two adult males were pooled before protein extraction to obtain a representative proteomic profile. Consequently, the analyzed seminal plasma and sperm fractions represented a single biological sample rather than biological or technical replicates.

### Animals

Two healthy adult male *T. aduncus*, aged 11 and 18 years and weighing approximately 110 and 105 kg, respectively, were included in the study. Both animals were maintained under identical environmental, nutritional, and reproductive management conditions. The dolphins were housed in outdoor seawater pools maintained at 24°C–32°C.

A single ejaculate was collected from each trained dolphin according to the method described by Fahrudin *et al.* [17]. During semen collection, each dolphin voluntarily positioned its ventral surface above the water level in response to trainer commands. Manual penile stimulation was then performed until ejaculation, and the semen was collected directly into a sterile collection bottle before being transported immediately to the laboratory. The semen characteristics of both individuals are presented in Table 1.

**Table 1:** Semen characteristics of the two Tursiops aduncus males.

**Characteristics**	**Male 1**	**Male 2**
Volume (mL)	26	29
Sperm concentration (×10⁶/mL)	1100	650
Total motility (%)	85.91	94.68
Viability (%)	85.70	94.70
Plasma membrane integrity (%)	62.6	72.6

Equal volumes of semen from the two dolphins were pooled and centrifuged at 4,000 × *g* for 30 min to separate the seminal plasma from the sperm pellet. Both fractions were stored at −20°C until protein extraction.

Only two adult male *T. aduncus* trained for semen collection were available in captivity in Indonesia. Because this study aimed to characterize the overall semen proteome rather than evaluate inter-individual variation, semen from both males was pooled before analysis. Therefore, the resulting seminal plasma and sperm samples represented a single biological sample, which precluded statistical comparisons.

### Protein extraction and sodium dodecyl sulfate-polyacrylamide gel electrophoresis separation

Sperm proteins were extracted using PRO-PREP™ Protein Extraction Solution (17081, iNtRON Biotechnology, Seongnam, South Korea). The sperm pellet was resuspended in the extraction solution. Before sodium dodecyl sulfate-polyacrylamide gel electrophoresis (SDS-PAGE), the total soluble protein concentrations of the seminal plasma and sperm fractions were determined using a bicinchoninic acid (BCA) protein assay kit (A65453, Thermo Fisher Scientific, Waltham, MA, USA). 

Proteins were separated according to molecular weight using SDS-PAGE, and protein bands were visualized after electrophoresis. Separation was performed on a SurePAGE™ Bis-Tris gel (10 × 8 cm, 12 wells, 4%–20% gradient; M00656, GenScript, Piscataway, NJ, USA) using Tris-MOPS-SDS running buffer (M00138; GenScript). Electrophoresis was performed at 200 V and 100–120 mA for 50 min. A Broad Multi Color Pre-Stained Protein Standard (5-270 kDa; M00624, GenScript) was used as the molecular weight marker.

Protein bands were excised from the gel, destained with 100 mM ammonium bicarbonate (A6141, Merck, Darmstadt, Germany) in 50% acetonitrile (A-0621-PB17, Fisher Chemical, Waltham, MA, USA), and dehydrated with 100% acetonitrile. The samples were dried at 30°C for 15 min using a SpeedVac (Thermo Fisher Scientific). Proteins were reduced with 10 mM dithiothreitol (3483-12-3, Sigma-Aldrich, St. Louis, MO, USA) in 100 mM ammonium bicarbonate for 30 min at 56°C and alkylated with 55 mM iodoacetamide (I1149, Sigma-Aldrich) in 100 mM ammonium bicarbonate for 30 min in the dark. After evaporation for 10 min, proteins were digested overnight at 37°C with sequencing-grade trypsin (10 μg/mL; V5111, Promega, Madison, WI, USA) prepared in 10 mM ammonium bicarbonate (A6141, Merck) containing 10% acetonitrile. Peptides were extracted with 10% formic acid and 70% acetonitrile at 5,000 × *g* for 30 min. The supernatant was evaporated to dryness in a SpeedVac (Eppendorf, Hamburg, Germany), resuspended in 1 mL MS-grade water (047146.Y0, Fisher Chemical), filtered through a 0.2 μm polyvinylidene fluoride membrane (88518, Thermo Fisher Scientific), and used for LC–MS/MS analysis.

## LC–MS/MS

Peptide separation was performed using a Vanquish™ Horizon ultra-high-performance liquid chromate-graphy system (Thermo Fisher Scientific, Bremen, Germany) equipped with an Acclaim™ PepMap™ 100 C18 column (150 × 1 mm, 3 μm; Thermo Fisher Scientific). The mobile phases consisted of MS-grade water containing 0.1% formic acid (A) and acetonitrile containing 0.1% formic acid (B), delivered at 75 μL/min. Gradient elution was initiated at 5% B, increased to 50% B over 31 min, and then returned to the initial conditions for column equilibration. The column temperature was maintained at 30°C, and the injection volume was 10 μL.

Mass spectrometric analysis was performed using an Orbitrap Exploris 240 high-resolution mass spectrometer (Thermo Fisher Scientific, Bremen, Germany). Data were acquired in full MS/dd-MS² mode under both positive and negative ionization conditions. Full MS spectra were collected at a resolution of 120,000 full width at half maximum over an *m/z* range of 350-1500, with a maximum injection time of 100 ms, an intensity threshold of 8,000, and charge states of 2-6. The precursor mass tolerance was set to 5 ppm. For dd-MS² acquisition, the resolution was 15,000 full-width at half maximum with a 2 *m/z* isolation window, normalized collision energy of 30, and nitrogen as the collision gas. The sheath, auxiliary, and sweep gas settings were 1, 5, and 1 arbitrary unit, respectively. The ion transfer tube temperature was maintained at 300°C, and the two most intense precursor ions were selected during each acquisition cycle.

### Protein profiling analysis

LC–MS/MS data were analyzed using Proteome Discoverer version 2.5 (Thermo Fisher Scientific, Waltham, MA, USA). Common contaminant proteins were excluded before downstream analyses. Trypsin was specified as the digestion enzyme, allowing up to one missed cleavage. Carbamidomethylation of cysteine was set as a fixed modification, whereas oxidation of methionine was specified as a variable modification. The precursor mass tolerance was set to 10 ppm, and protein identifications were accepted at a false discovery rate (FDR) of 1%.

Proteins were identified using the UniProt *T. truncatus* protein database. Only proteins identified with at least two unique peptides and a High Troughput score >0 were retained for further analysis. GO enrichment and protein-protein interaction analyses were performed using Search Tool for the Retrieval of Interacting Genes/Proteins (STRING) version 12.0 (https://string-db.org/) with the *Tursiops* background database. Statistical significance was defined as an FDR of <0.05 with a confidence score >0.4.

### Statistical analysis

Statistical analyses were not performed because semen samples from the two dolphins were pooled before protein analysis. Consequently, the resulting measurements represented a single biological sample rather than independent biological replicates, making statistical comparisons inappropriate.

## RESULTS

### Protein identification

**Figure 1:**
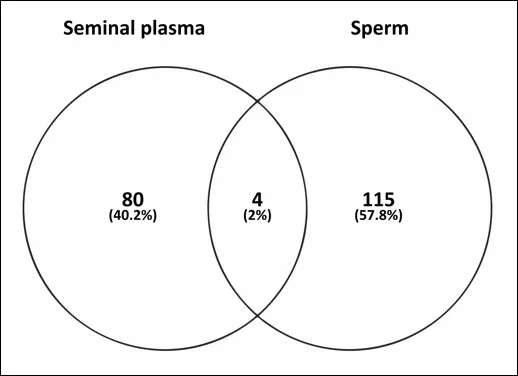
Venn diagram showing the numbers of proteins uniquely identified in seminal plasma (80), uniquely identified in sperm (115), and shared between seminal plasma and sperm (4) in *Tursiops aduncus*.

A total of 199 proteins were identified in this study. Of these, 84 proteins were detected in seminal plasma and 119 in sperm, with only four proteins, namely aldolase C (ALDOC), basigin (BSG), creatine kinase B-type (CKB), and lactate dehydrogenase A (LDHA), shared between the two fractions. Consequently, 80 proteins were unique to seminal plasma and 115 were unique to sperm (Figure 1). Complete lists of the identified proteins are provided in Supplementary Tables S1 (seminal plasma) and S2 (sperm).

### GO analysis

**Figure 2:**
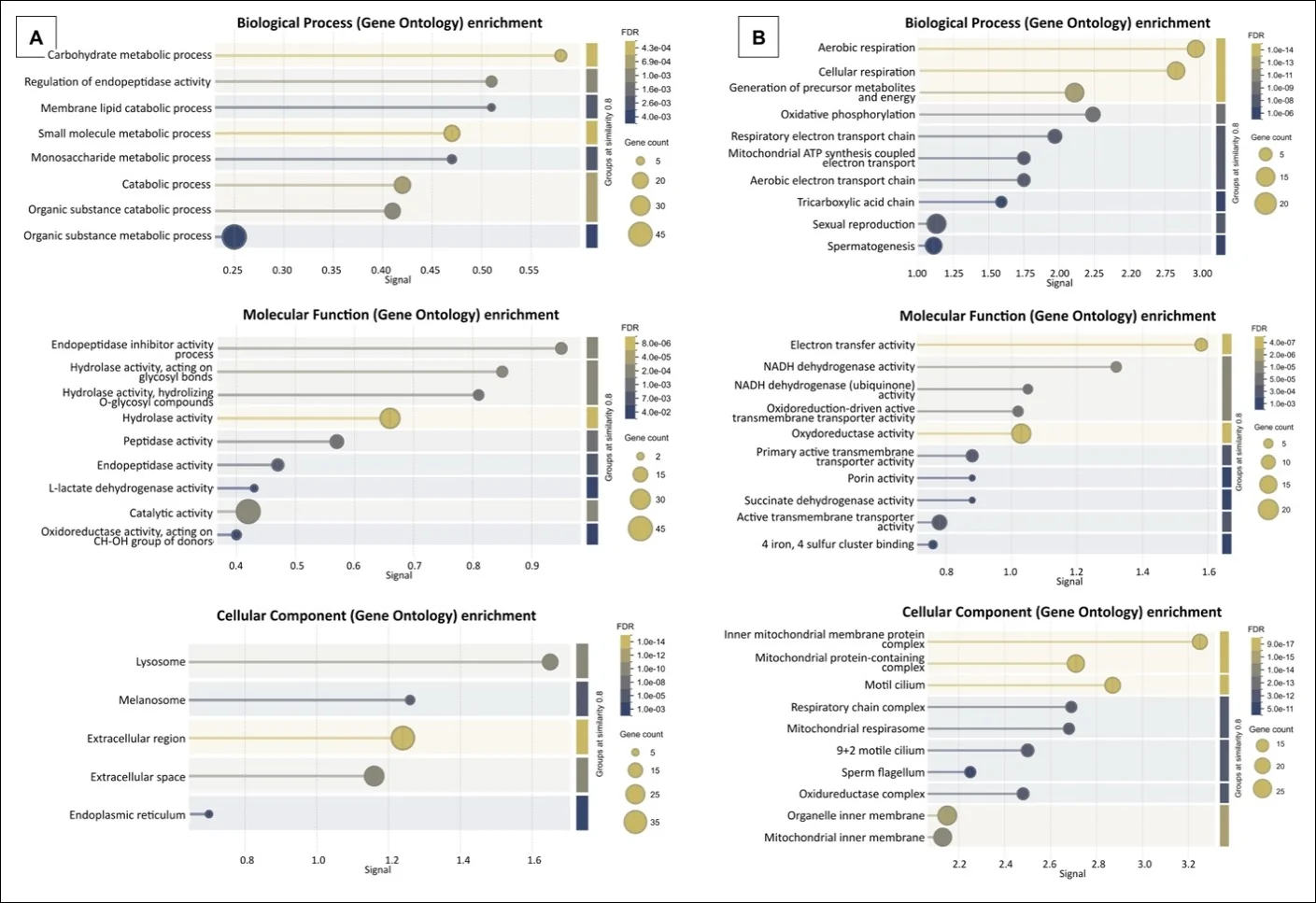
GO enrichment analysis of proteins identified in seminal plasma (A) and sperm (B) of *T. aduncus*. Proteins were classified according to biological processes, molecular functions, and cellular components. Only significantly enriched GO terms (FDR < 0.05) are shown. GO = Gene Ontology; FDR = False discovery rate; *T. aduncus* = *Tursiops aduncus*.

GO analysis classified the identified proteins into the categories of biological process, molecular function, and cellular component (Figure 2).

Within the biological process category, seminal plasma proteins were enriched in carbohydrate metabolic process (GO:0005975), catabolic process (GO:0009056), small molecule metabolic process (GO:0044281), monosaccharide metabolic process (GO:0005996), organic substance catabolic process (GO:1901575), membrane lipid catabolic process (GO:0046466), and regulation of endopeptidase activity (GO:0052548). In contrast, sperm proteins were enriched in cellular respiration (GO:0045333), aerobic respiration (GO:0009060), oxidative phosphorylation (GO:0006119), respiratory electron transport chain (GO:0022904), mitochondrial adenosine triphosphate (ATP) synthesis-coupled electron transport (GO:0042775), aerobic electron transport chain (GO:0019646), tricarboxylic acid cycle (GO:0006099), sexual reproduction (GO:0019953), and spermatogenesis (GO:0007283).

For the molecular function category, seminal plasma proteins were enriched in endopeptidase inhibitor activity (GO:0004866), hydrolase activity acting on glycosyl bonds (GO:0016798), peptidase activity (GO:0008233), endopeptidase activity (GO:0004175), oxidoreductase activity (GO:0016614), and L-lactate dehydrogenase activity (GO:0004459). In contrast, sperm proteins were enriched in electron transfer activity (GO:0009055), Nicotinamide adenine dinucleotide (NADH) dehydrogenase activity (GO:0003954), NADH dehydrogenase (ubiquinone) activity (GO:0008137), oxidoreduction-driven active transmembrane transporter activity (GO:0015453), primary active transmembrane transporter activity (GO:0015399), porin activity (GO:0015288), active transmembrane transporter activity (GO:0022804), and succinate dehydrogenase activity (GO:0000104).

Within the cellular component category, seminal plasma proteins were enriched in lysosome (GO:0005764), extracellular region (GO:0005576), extracellular space (GO:0005615), and endoplasmic reticulum lumen (GO:0005788). In contrast, sperm proteins were enriched in mitochondrial inner membrane (GO:0005743), inner mitochondrial membrane protein complex (GO:0098800), mitochondrial protein-containing complex (GO:0098798), mitochondrial respirasome (GO:0005746), motile cilium (GO:0031514), 9+2 motile cilium (GO:0097729), and sperm flagellum (GO:0036126).

### Protein-protein interaction network

**Figure 3:**
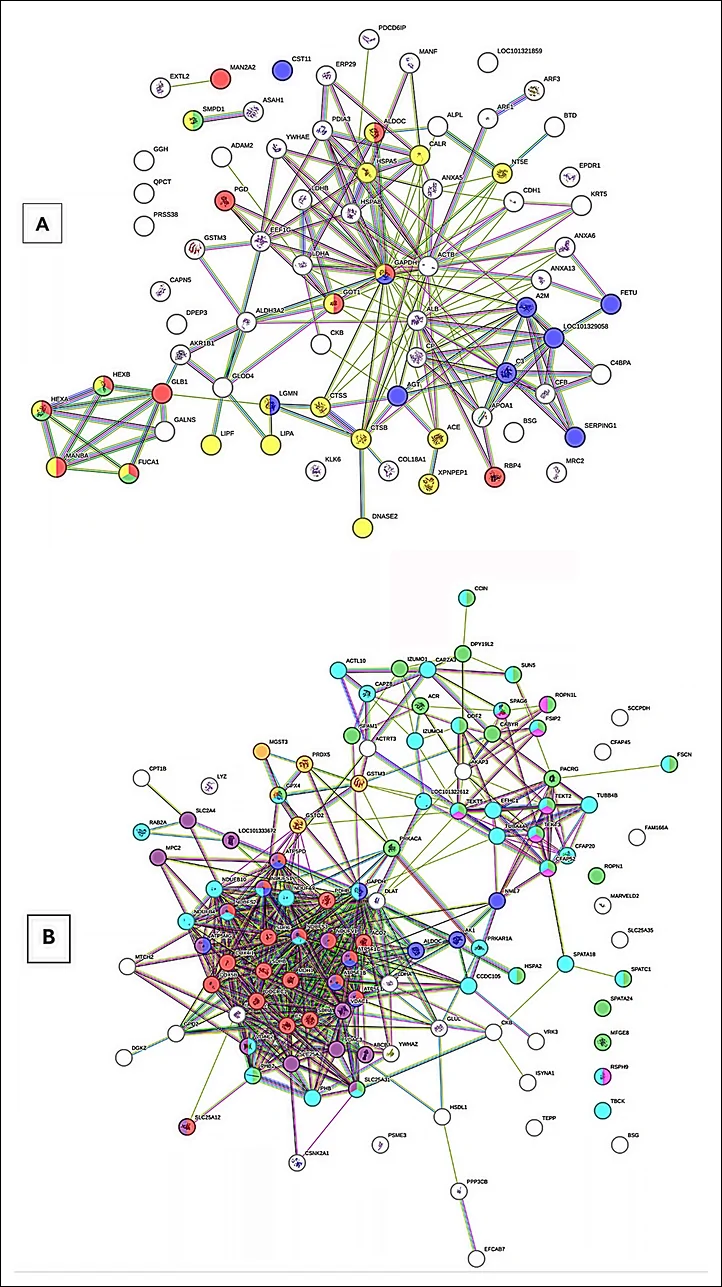
STRING PPI networks of seminal plasma proteins (A) and sperm proteins (B) from *T. aduncus*. The seminal plasma network identified ALB, GAPDH, and ACTB as the major hub proteins, whereas the sperm network identified ATP5F1C and UQCRC2 as the major hub proteins. The seminal plasma network comprised 72 nodes and 173 edges (expected edges = 43; PPI enrichment p < 1.0 × 10⁻¹⁶), whereas the sperm protein network comprised 103 nodes and 491 edges (expected edges = 101; PPI enrichment p < 1.0 × 10⁻¹⁶). STRING = Search Tool for the Retrieval of Interacting Genes/Proteins; PPI = Protein-protein interaction; ALB = Serum albumin; GAPDH = Glyceraldehyde-3-phosphate dehydrogenase; ACTB = Beta-actin; ATP5F1C = ATP synthase F1 subunit gamma; UQCRC2 = Ubiquinol-cytochrome c reductase core protein 2; *T. aduncus* = *Tursiops aduncus*.

Protein-protein interactions were analyzed using STRING (Figure 3). The seminal plasma proteins formed a relatively dispersed interaction network, whereas the sperm proteins exhibited a more interconnected network. In the seminal plasma network, beta-actin (ACTB), serum albumin (ALB), and glyceraldehyde-3-phosphate dehydrogenase (GAPDH) had the highest interaction scores (Figure 3A). In contrast, the sperm protein network comprised several interconnected clusters associated with cellular respiration, intracellular structural organization, cell motility, and reproductive processes (Figure 3B).

## DISCUSSION

### Proteomic characteristics of dolphin semen

This study provides the first comprehensive proteomic characterization of seminal plasma and sperm from *T. aduncus*. The findings establish a species-specific molecular reference and expand the current understanding of cetacean reproductive biology. Although the seminal plasma and sperm proteomes of *T. truncatus* have previously been characterized [16], corresponding proteomic information for *T. aduncus* has not been reported.

Dolphin seminal plasma originates primarily from the testes, epididymides, and prostate, the only accessory sex gland described in cetaceans [11]. A total of 199 proteins were identified in the seminal plasma and sperm fractions. Only four proteins were shared between the two fractions: aldolase C, fructose-bisphosphate (ALDOC), basigin (BSG), creatine kinase B-type (CKB), and L-lactate dehydrogenase (LDHA). ALDOC and LDHA have also been detected in both seminal plasma and sperm of *T. truncatus* [16].

ALDOC is involved in sperm energy metabolism, including phosphocreatine and ATP synthesis, as well as NAD/NADH metabolism [18]. LDHA contributes to sperm function and fertility [19]. Both proteins are localized in the fibrous sheath of spermatozoa, where they anchor metabolic enzymes along the flagellum and provide a localized adenosine triphosphate (ATP) supply required for sperm motility [20].

BSG belongs to the immunoglobulin superfamily and is required for both male and female reproduction. It is initially localized in the principal piece of testicular spermatozoa, relocates to the midpiece during epididymal maturation, and subsequently moves to the sperm head after capacitation, indicating its role in sperm-zona pellucida interaction [21].

CKB rapidly regenerates ATP from adenosine diphosphate (ADP) using phosphocreatine, thereby supporting efficient energy generation, transport, and utilization in spermatozoa [22]. Phosphorylation of CKB by protein kinase C further enhances its enzymatic activity by facilitating ATP regeneration.

### Seminal plasma proteins

GO analysis showed enrichment of the organic substance catabolic process in seminal plasma. Proteins associated with this process included Beta-hexosaminidase (HEXA and HEXB), Alpha-L-fucosidase (FUCA1), Beta-mannosidase (MANBA), Deoxyribonuclease-2-alpha (DNASE2), Cathepsin B (CTSB), Cathepsin S (CTSS), Legumain (LGMN), Lipase (LIPA and LIPF), angiotensin-converting enzyme (ACE), Xaa-Pro aminopeptidase 1 isoform X1 (XPNPEP1), Glyceraldehyde-3-phosphate (GAPDH), Aspartate aminotransferase (GOT1), Calreticulin (CALR), Endoplasmic reticulum chaperone BIP (HSPA5), 5’-nucleotidase (NT5E), Sphingomyelin phosphodiesterase (SMPD1), and ALDOC.

These proteins participate in the degradation and processing of proteins, lipids, and nucleic acids that may influence the post-ejaculatory environment of spermatozoa. This finding is consistent with the enrichment of GO molecular function terms associated with endopeptidase and peptidase activities. Protease regulation can occur without new protein synthesis, making it suitable for controlling extracellular processes, including interactions between seminal plasma proteins and spermatozoa, as well as between spermatozoa and the female reproductive tract. However, proteolysis must be tightly regulated because it also contributes to remodeling sperm surface proteins during preparation for capacitation while preventing premature activation and tissue damage [23].

Membrane lipid catabolism was also enriched in seminal plasma. Plasma membrane lipids are essential for maintaining the structural integrity and physiological functions of mammalian spermatozoa. Coordinated lipid metabolism contributes to sperm motility, capacitation, the acrosome reaction, and sperm-oocyte fusion. Consequently, sperm fertility depends greatly on maintaining lipid homeostasis [24].

Carbohydrate metabolism was also enriched in seminal plasma and involved HEXA, HEXB, FUCA1, MANBA, beta-galactosidase (GLB1), retinol-binding protein (RBP4), GAPDH, 6-phosphogluconate dehydrogenase (PGD), alpha-mannosidase (MAN2A2), GOT1, and ALDOC. Although proteins associated with carbohydrate metabolism were enriched in seminal plasma, previous studies have shown that dolphin spermatozoa maintain motility primarily through β-oxidation of fatty acids rather than glycolysis [25].

Several seminal plasma proteins may also help protect spermatozoa against oxidative stress. HSPA5 functions as a molecular chaperone that protects cells by enhancing antioxidant synthesis, stabilizing proteins, and activating compensatory energy pathways [26]. GAPDH is an essential glycolytic enzyme that is susceptible to oxidative inactivation, thereby redirecting glucose metabolism toward the pentose phosphate pathway. This metabolic shift reduces NADH production through glycolysis while increasing NADPH production through the pentose phosphate pathway, contributing to short-term adaptation to oxidative stress [27]. RBP4 is the principal transport protein for retinol (vitamin A) [28] and has also been identified previously in the seminal plasma of *T. truncatus* [16].

Collectively, the identified proteins, enriched GO terms, and protein-protein interaction network suggest that seminal plasma provides a supportive extracellular environment that promotes sperm function and protection. This observation is consistent with previous reports demonstrating that seminal plasma provides nutrients, protects spermatozoa, acts as a buffering medium, and supports sperm motility [29].

### Spermatozoa proteins

GO biological process enrichment analysis of spermatozoa proteins revealed enrichment of terms associated with energy production, including oxidative phosphorylation (GO:0006119), aerobic respiration (GO:0009060), proton motive force-driven mitochondrial ATP synthesis (GO:0042776), proton motive force-driven ATP synthesis (GO:0015986), respiratory electron transport chain (GO:0022904), and mitochondrial ATP synthesis-coupled electron transport (GO:0042775). Collectively, these enriched GO terms indicate that energy production in dolphin sperm is predominantly supported by aerobic respiration through oxidative phosphorylation.

The proteomic findings further support the distinctive metabolic characteristics previously described for dolphin sperm [25, 30]. Unlike humans and rodents, which rely primarily on glycolysis, and ruminants and canines, which use both glycolysis and oxidative phosphorylation for energy production [31], dolphin sperm depend predominantly on endogenous fatty acid oxidation as an adaptation to the carbohydrate-limited marine environment [25]. Although glycolysis-associated proteins such as ALDOC and LDHA were detected in seminal plasma, they may originate from somatic cells of the male reproductive tract or extracellular secretions rather than reflecting active glycolytic metabolism in spermatozoa. In contrast, enrichment of oxidative phosphorylation, respiratory electron transport chain, mitochondrial ATP synthesis, and proton motive force-driven ATP synthesis pathways supports the importance of mitochondrial ATP production in delphinid sperm [25, 30]. The substrates for oxidative phosphorylation are likely derived from the high fatty acid content previously reported in dolphin sperm. Fatty acids are transported into mitochondria through the carnitine shuttle, which depends on Carnitine O-palmitoyltransferase 1, muscle isoform (CPT1A), and subsequently undergo β-oxidation to generate acetyl-CoA. Acetyl-CoA then enters the tricarboxylic acid cycle, producing NADH and FADH₂, which supply electrons to the mitochondrial electron transport chain and drive oxidative phosphorylation [25]. These findings are consistent with fatty acid β-oxidation serving as the principal energy source supporting sperm function in delphinids.

Other enriched GO biological processes were associated with flagellated sperm motility (GO:0030317), spermatogenesis (GO:0007283), spermatid development (GO:0007286), cilium-dependent cell motility (GO:0060285), cilium movement involved in cell motility (GO:0060294), and sperm capacitation (GO:0048240). These processes involved proteins including Ropporin-1-like protein (ROPN1L), Sperm-associated antigen 6 isoform X1 (SPAG6), Calcium-binding tyrosine phosphorylation-regulated protein (CABYR), Fibrous sheath-interacting protein 2 (FSIP2), Parkin coregulated gene protein (PACRG), Tektin (TEKT2, TEKT3, and TEKT5), Tubulin beta chain (TUBB4B), cAMP-dependent protein kinase catalytic subunit alpha (PRKACA), and Glutathione peroxidase (GPX4). Several proteins identified in the present study, including A-kinase anchor protein 3 (AKAP3), outer dense fiber protein 2 (ODF2), TUBB, GSTM3, and ROPN1, have also been reported in the sperm proteome of *T. truncatus* [16].

AKAP3 is an essential structural component of the fibrous sheath and plays a critical role in sperm flagellar formation, motility, and fertility [32]. The fibrous sheath is a sperm-specific cytoskeletal structure surrounding the outer dense fibers and axoneme that contributes to the regulation of flagellar movement. Loss of AKAP3 causes widespread alterations in the sperm proteome, mislocalization of sperm proteins, and dysregulation of protein kinase A (PKA) activity [32]. ROPN1 and ROPN1L bind to AKAP3 and function cooperatively to maintain its incorporation into the fibrous sheath. Simultaneous deficiency of both proteins reduces cAMP-dependent protein kinase phosphorylation and capacitation-associated tyrosine phosphorylation, resulting in impaired fertility [33]. Reduced abundance of these proteins has also been associated with poor blastocyst development [34].

SPAG6 is a component of the central apparatus of the “9 + 2” axoneme and is essential for ciliary and flagellar motility. In addition to its structural role, SPAG6 functions as a chaperone during spermatogenesis, and its deficiency disrupts meiosis and causes morphological abnormalities of the acrosome, manchette, and flagella [35, 36]. ODF2 is a major component of the outer dense fibers of the sperm flagellum and is initially stored in the granulated body of post-meiotic spermatids before being incorporated into the sperm tail during elongation [37]. ODF2 contributes to flagellar stability and sperm transport, whereas its deficiency causes separation of the neck and midpiece [38]. ODF proteins stabilize the axoneme, and their reduced expression has been associated with asthenozoospermia [39], whereas greater abundance has been associated with improved fertility and semen quality [40].

### CABYR, tubulin, tektins, and detoxification-associated proteins

CABYR is localized to the fibrous sheath, an accessory structure of the sperm flagellar principal piece. Functional studies have demonstrated that CABYR knockout results in severe subfertility characterized by impaired sperm motility, marked disorganization of the fibrous sheath, and abnormal doublet microtubule configuration [41]. In addition, CABYR is required to maintain sperm chirality, a process influenced by intracellular Ca²⁺ homeostasis [42]. CABYR also interacts with AKAP3 and ROPN1 to facilitate the assembly of signaling complexes within the fibrous sheath [43]. These findings highlight the importance of CABYR in maintaining the structural integrity and functional organization of the sperm flagellum.

The tubulin proteins identified in this study, TUBA4A and TUBB4B, are essential cytoskeletal components involved in microtubule organization and the development of cilia and flagella [44]. However, increased TUBA4A expression has also been reported in sperm from men with asthenozoospermia compared with normozoospermic controls [45]. Tektins are a family of microtubule-stabilizing proteins indispensable for the assembly and structural stability of cilia and flagella. Deficiency of tektin family members disrupts microtubule integrity and impairs sperm motility [46]. TEKT3 and TEKT4 are enriched in male germ cells, and TEKT4 is essential for coordinated and progressive sperm motility in mice. Male mice lacking TEKT3 produce sperm with reduced motility, diminished forward progression, and increased flagellar bending defects [46]. TEKT2 has also been reported to be downregulated in teratozoospermia and is essential for ciliary and flagellar development [47]. TEKT5 is expressed exclusively in the testes. Collectively, tektins play indispensable roles in sperm motility and male fertility. Deficiency of TEKT2 or TEKT4 results in infertility or subfertility, whereas loss of TEKT3 reduces sperm motility and forward progression. Furthermore, the organization of TEKT1, TEKT2, and TEKT4 filaments is highly conserved among sea urchin sperm, mammalian sperm, and mammalian respiratory cilia [48].

Several identified proteins are also associated with cellular detoxification and protection against oxidative stress. GSTM3, a member of the glutathione S-transferase family, catalyzes the conjugation of electrophilic compounds to reduced glutathione and contributes to cellular antioxidant defense. GSTM3 has also been proposed as a potential biomarker of male infertility or subfertility and is distributed throughout the midpiece, principal piece, and end piece of the sperm tail [49]. PRDX5 provides additional antioxidant protection and is localized to the acrosomal cap and post-acrosomal region [50]. Together, these proteins may help preserve sperm function by protecting spermatozoa from oxidative damage.

The identification of highly connected proteins, including AKAP3, ODF2, SPAG6, and Glutathione S-transferase Mu 3 (GSTM3), suggests that these molecules may serve as candidate biomarkers for non-invasive assessment of male fertility in captive and wild dolphin populations.

Compared with the *T. truncatus* seminal proteome reported by Fuentes-Albero *et al.* [16], the overall number of proteins identified differed substantially. Nevertheless, the five most abundant sperm proteins reported in that study (AKAP3, ODF2, TUBB, GSTM3, and ROPN1) were also identified in the present study, indicating conservation of core structural and motility-associated proteins across *Tursiops* species. In contrast, the five most abundant seminal plasma proteins reported by Fuentes-Albero *et al.* [16] (CST11, LTF, ALB, HSP90B1, and PIGR) differed from the proteins with the highest interaction scores identified in the seminal plasma of *T. aduncus* (ACTB, ALB, and GAPDH), with ALB being the only protein common to both datasets. This difference may reflect species-specific variation in seminal plasma composition, although methodological differences between studies cannot be excluded. Because a species-specific *T. aduncus* reference proteome is currently unavailable, protein identification in the present study relied on the UniProt and STRING databases for *T. truncatus*. This database limitation should be considered when interpreting potential species-specific proteins and underscores the need to develop a dedicated *T. aduncus* reference proteome for future proteomic investigations.

### Limitation

This study has several limitations. First, the proteomic analysis used a single pooled semen sample from two adult *T. aduncus*, which limited statistical power, precluded assessment of inter-individual biological variation, and prevented formal quantitative comparisons of protein abundance between seminal plasma and sperm. Second, protein identification relied on the *T. truncatus* reference database because a dedicated *T. aduncus* proteome is not currently available, which may have introduced bias in identifying species-specific proteins. Third, the candidate proteins identified in this study were not validated using orthogonal techniques, such as Western blotting or immunofluorescence, and their associations with fertility were not evaluated. Future studies should include larger sample sizes with individual-level analyses, quantitative proteomic approaches, functional validation of candidate biomarkers, and correlation of protein expression with reproductive performance.

## CONCLUSION

This study provides the first comprehensive proteomic characterization of seminal plasma and sperm from *T. aduncus*, identifying 199 proteins with distinct functional profiles between the two fractions. Seminal plasma was enriched in proteins associated with organic substance catabolism, lipid and carbohydrate metabolism, and antioxidant defense, highlighting its supportive role in maintaining the extracellular environment required for sperm survival and function. In contrast, sperm proteins were predominantly enriched in biological processes related to oxidative phosphorylation, mitochondrial ATP production, flagellar motility, spermatogenesis, and sperm capacitation, consistent with the specialized energy metabolism and structural adaptations of dolphin sperm. The identification of key proteins involved in energy production (e.g., ALDOC, LDHA, and CKB), flagellar organization (e.g., AKAP3, ODF2, SPAG6, CABYR, and tektins), and antioxidant protection (e.g., GSTM3 and PRDX5) provides molecular insights into the mechanisms supporting sperm function in this marine mammal.

A major strength of this study is the generation of the first proteomic resource for *T. aduncus* semen, providing a valuable reference for future studies of cetacean reproductive biology. Despite the limited sample size, integrating proteomic profiling, GO enrichment, and protein-protein interaction analyses enabled comprehensive functional characterization of seminal plasma and sperm proteins and facilitated comparison with the available proteome of *T. truncatus*.

These findings have practical implications for dolphin conservation and reproductive management by providing candidate molecular markers that may help evaluate semen quality, reproductive function, and the development of assisted reproductive technologies for captive breeding and conservation programs. Future studies should include larger cohorts, quantitative proteomic analyses, species-specific protein databases, and functional validation of candidate biomarkers, together with correlations between protein expression and reproductive performance, to establish reliable molecular indicators of male fertility in cetaceans.

Overall, this study expands the current understanding of the molecular composition and functional organization of dolphin semen and establishes a foundation for future investigations into reproductive physiology, fertility assessment, and conservation of *T. aduncus*.

## DATA AVAILABILITY

### The supplementary data are available from the corresponding author upon request. 

## GENERATIVE AI DECLARATION

The authors declare that generative artificial intelligence (AI) tools were used solely to improve language, grammar, and readability during manuscript preparation. The authors developed and verified all scientific content, data analysis, interpretation of results, and conclusions.

## AUTHORS’ CONTRIBUTIONS

MF, NWK, and IA: Conceived and designed the study. MF, BWW, and WEP: Collected the data. MF, NWK, IA, BWW, and WEP: Analyzed and interpreted the data. MF: Drafted the manuscript. NWK and IA: Supervised the study. AI: Collected samples and critically reviewed the manuscript. All authors have read and approved the final manuscript.
